# Estimating fermentation characteristics and nutritive value of ensiled and dried pomegranate seeds for ruminants using *in vitro* gas production technique

**Published:** 2012-05-12

**Authors:** M. Taher-Maddah, N. Maheri-Sis, R. Salamatdoustnobar, A. Ahmadzadeh

**Affiliations:** *Department of Animal Science, Shabestar Branch, Islamic Azad University, Shabestar, Iran*

**Keywords:** Drying, Ensiling, Gas production, Nutritive value, Pomegranate seeds

## Abstract

The purpose of this study was to determine the chemical composition and estimation of fermentation characteristics and nutritive value of ensiled and dried pomegranate seeds using *in vitro* gas production technique. Samples were collected, mixed, processed (ensiled and dried) and incubated *in vitro* with rumen liquor taken from three fistulated Iranian native (Taleshi) steers at 2, 4, 6, 8, 12, 16, 24, 36, 48, 72 and 96 h. The results showed that ensiling lead to significant increase in gas production of pomegranate seeds at all incubation times. The gas volume at 24 h incubation, were 25.76 and 17.91 ml/200mg DM for ensiled and dried pomegranate seeds, respectively. The gas production rate (*c*) also was significantly higher for ensiled groups than dried (0.0930 vs. 0.0643 ml/h). The organic matter digestibility (OMD), metabolizable energy (ME), net energy for lactation (NEL) and short chain fatty acids (SCFA) of ensiled pomegranate seeds were significantly higher than that of dried samples (43.15%, 6.37 MJ/kg DM, 4.43 MJ/kg DM, 0.5553 mmol for ensiled samples vs. 34.62%, 5.10 MJ/kg DM, 3.56 MJ/kg DM, 0.3680 mmol for dried samples, respectively). It can be concluded that ensiling increases the nutritive value of pomegranate seeds.

## Introduction

Using agro-industrial by-products is an alternative method for overcoming shortages and higher prices of conventional feed in ruminant nutrition. Many by-products have a substantial nutritive value as animal feed. Thus cereals can be largely replaced by these by-products and therefore competition between human and animal for nutrition is less. Furthermore, using these by-products in animal nutrition can solve related environmental problems (Mirzaei-Aghsaghali and Maheri-Sis *et al.*, 2008).

Pomegranate (*Punica granatum* L.) is an important fruit crop in tropical and subtropical regions of the world as well as in Mediterranean countries with moderate temperatures. The pomegranate is native to Iran with 7×10^5^ tons produced annually (Khoshnam *et al.*, 2007; Mansouri *et al.*, 2011). Iran is also a major exporter of pomegranates, with an increase from 14,075 metric tons (2003) to 27,439 metric tons (2007) (FAO/WHO, 2009).

This fruit is either consumed fresh or used in the juice industry. Increasing agro-industrial units for producing pomegranate juice led to increased processing of by-products including peels and seeds (Shabtay *et al.*, 2008). Pomegranate seeds constitute about 3% of the weight of the fresh fruit. The major chemical components of pomegranate seeds are: 1) Hydroxybenzoic acids: Ellagic acid, 3,3’-Di-O-methylellagic acid, 3,3’.4’-Tri-O-methylellagic acid; 2) Conjugated fatty acids: Punicic acid (cis-9, trans-11, cis-13 octadecatrienoic acid); 3) Non-Conjugated fatty acids: Linoleic acid, Oleic acid, Palmitic acid, Stearic acid; 4) Sterols: Stigmasterol, β-Sitosterol, Daucosterol, Camesterol, Cholesterol, 17-α-Estradiol, Estrone, Testosterone, Estriol; 5) Tocopherols: γ-tocopherol; 6) Triterpenes: Ursolic acid, Oleanolic acid; 7) Isoflavones: Genistein, Daidzein; 8) Phenyl aliphatic glycosides/Lignins: Coniferyl-9-O-[β-Dapiofuranosyl (1→6)-O-β-D-glucopyranoside, Sinapyl-9-O-[β- D-apiofuranosyl (1→6)-O-β-D-glucopyranoside, Phenylethyl rutinoside, Icariside D1 (Prakash and Prakash, 2011).

Pomegranate seed oil comprises 12–20% of total seed weight. The oil consists of about 80% conjugated octadecatrienoic fatty acids, with a high content of *cis 9, trans 11, cis 13* acid (i.e. punicic acid), synthesized from nonconjugated octadecadienoic fatty acid. Linoleic acid which is the most important essential fatty acid, constitute about 7% of pomegranate seed oil.

The presence of these components in pomegranate seed, make it a functional food and is believed to be beneficial to health by acting as an antioxidant as well as having anti-cancer and anti-inflammatory properties (Lansky and Newman, 2007).

In spite of sufficient knowledge of the biological effects of pomegranate seeds in human and animal health, there is scant information on its nutritive value for ruminants (Feizi *et al.*, 2005; Shabtay *et al.*, 2008; Modarresi *et al.*, 2010; Mirzaei-Aghsaghali *et al.*, 2011). Feizi *et al.*, (2005) demonstrated that pomegranate seeds can be used in animal nutrition and that inclusion of pomegranate seeds up to 25% of the diet had no negative effect on the intake and digestibility of nutrients.

Shabtay *et al.*, (2008) reported that using pomegranate by-products in levels up to 20% of dietary DM in feedlot calves diet increased feed intake and consequently average daily weight gain.

Modarresi *et al.*, (2010) concluded that pomegranate seed pulp is an inexpensive source of feed and can be replaced with part of energy rich feed for goats, such as cereal grains. Mirzaei-Aghsaghali *et al*. (2011) suggested that pomegranate seed can be used as a relatively good agro-industrial by-product for ruminant nutrition.

Pomegranate seed pulp can be used in fresh, dried and ensiled forms in animal nutrition. Due to its potential for rapid spoiling it is usually preserved.

Drying and ensiling are two common preservation methods of wet feeds for further use. Each of these methods has distinct advantages and disadvantages. Due to the environmental conditions in which pomegranates are harvested, sun-drying is difficult, therefore ensiling is the preferred method.

Among routine feed evaluation methods (*in vivo, in situ* and *in vitro*), *in vitro* gas production technique is a cost and time effective tool for illustration of fermentation kinetics and estimating nutritive value of feedstuffs, particularly for polyphenolic content in feed in developing countries (Makkar, 2005; Maheri-Sis *et al.*, 2007).

The objectives of this study were to determine chemical composition and estimating fermentation parameters [*a*: the gas production from soluble fraction (ml/200mg DM), *b*: the gas production from insoluble but fermentable fraction (ml/200mg DM), *c*: rate constant of gas production during incubation (ml/h)] and nutritive value [organic matter digestibility (OMD), metabolizable energy (ME), net energy for lactation (NEL) and short chain fatty acids (SCFA)] of dried and ensiled pomegranate seeds using *in vitro* gas production technique.

## Methods and Material

### Samples collection and treatments

Fresh pomegranate seeds samples (after juice extracting; with no any other parts of pomegranate by-products such as peels) were collected from traditional (local) pomegranate juice production units, in Tehran, Iran. After mixing, half of the samples were air-dried (7 days (d) at room temperature i.e. about 25 degrees Celcius) and ground (1mm and 5mm screen).

The remaining samples were ensiled in P.V.C tubes for 45 d. Chemical analysis and *in vitro* gas production were conducted at the laboratory of Animal Science Research Institute in Karaj, Iran.

### Chemical Analysis

Dry matter (DM) was determined by drying the samples at 105°C overnight and reduced to ash by igniting (burning) the samples in a muffle furnace at 525°C for 8h. Nitrogen (N) content was measured by the Kjeldahl method (AOAC, 1990). Crude protein (CP) was calculated as N × 6.25. Neutral detergent fiber (NDF) and acid detergent fiber (ADF) were determined by procedures outlined by Van Soest *et al*. (1991). Non-fibrous carbohydrate (NFC) is calculated using the equation of NRC (2001); NFC = 100 – (NDF + CP + EE + Ash).

### In vitro gas production procedure

Fermentation of dried and ensiled pomegranate seed samples were carried out with rumen fluid obtained from three fistulated Iranian native steers (Taleshi) fed twice daily a diet containing alfalfa hay (60%) and concentrate mixture (40%) following the method described by Menke and Steingass (1988). Both solid and liquid rumen fractions were collected before the morning feeding, placed in an insulated plastic container, sealed immediately and transported to the laboratory.

Approximately 200 mg (DM) of each sample was weighed into 100 ml glass syringes. The fluid-buffer mixture (30 ml) was transferred into 100 ml glass syringes. The glass syringes containing samples and rumen fluid-buffer mixture were subsequently incubated at 39°C. The syringes were gently shaken 30 min after the start of incubation. The gas production was determined at 2, 4, 6, 8, 12, 24, 48, 72 and 96 h of incubation. All samples were incubated in triplicate with three syringes containing only rumen fluid-buffer mixture (blank).

The net gas productions of samples were determined by subtracting the volume of gas produced in the blanks. Gas production data were fitted to the model of Ørskov and McDonald (1979):

*Y* = *a* + *b* (1-e^-*ct*^)

Where *Y* is the gas production at time *t*, *a* the gas production from soluble fraction (ml/200mg DM), *b* the gas production from insoluble but fermentable fraction (ml/200mg DM), *c* the gas production rate constant (ml/h), *a* + *b* the potential gas production (ml/200mg DM) and *t* is the incubation time (h).

The ME, NEL and OMD of pomegranate seeds were calculated using equations of Menke and Steingass (1988) as:

ME (MJ /kg DM) = 2.20 + 0.136 × GP + 0.057 × CP

NEL (MJ/kg DM) = 0.115 × GP + 0.0054 × CP + 0.014 × EE - 0.0054 × CA - 0.36

OMD (g/kg DM) = 14.88 + 0.889 × GP + 0.45 × CP + 0.0651 × CA

Where, GP is 24 h net gas production volume (ml/200 mg DM), and CP, EE, CA are crude protein, ether extract and crude ash (g/kg DM), respectively.

Short chain fatty acids (SCFA) are calculated by equation of Getachew *et al*. (1999):

SCFA (mmol) = -0.0601 + (0.0239 × GP)

Where, GP is 24 h net gas production volume (ml/200mg DM).

### Statistical analysis

All of the data obtained from three replicates were analyzed using software of SAS (1991) and the means of two sample groups were separated by independent-samples t-test (McDonald, 2008).

## Results and Discussion

The chemical analysis of ensiled and dried pomegranate seeds (Table 1) showed that NDF, ADF, EE and ash content of pomegranate seeds decreased and NFC and CP content were increased by ensiling. In other words, dried seeds have higher fiber components (cellulose, hemicelluloses and lignin) than ensiled forms.

**Table 1:** Chemical composition of dried and ensiled pomegranate seeds (%)

Items	Dried	Ensiled
DM	96.61	42.76
CP	8.07	11.65
EE	12.00	11.00
Ash	2.80	2.05
NDF	77.00	62.00
ADF	55.00	45.00
NFC	0.13	13.30

DM: dry matter, CP: crude protein, EE: ether extract, NDF: neutral detergent fiber, ADF: acid detergent fiber, NFC: non fibrous carbohydrate.

Lansky and Newman (2007) stated that pomegranate seed matrix includes lignin, fusion products of cell wall components and hydroxycinnamic acids, and potently antioxidant lignin derivatives.

Maheri-Sis (2010) indicated that about 50% of hemicelluloses of ensiled plants may be degraded during ensiling process. Degradation of hemicelluloses content can result in lower NDF and ADF content of ensiled materials.

Chemical compositions of dried pomegranate seeds in the current study were inconsistent with findings of Feizi *et al.*, (2005) and Mirzaei-Aghsaghali *et al.*, (2011). According to Feizi *et al.*, (2005) the amount of DM, OM, CP, crude fiber (CF), EE, nitrogen free extract (NFE) of pomegranate seeds were 94.8, 96.8, 11.4, 38.9, 1, and 45.5, respectively; whereas Mirzaei-Aghsaghali *et al.*, (2011) reported that DM, CP, EE, NDF, ADF and NFC content of pomegranate seeds were 95.10, 15.40, 6.00, 68.00, 49.00 and 13.50%, respectively.

These differences in chemical composition of by-products may be due to a difference in starting materials, varieties, growing conditions (i.e. geographic, climatic and soil characteristics), amount of foreign materials and impurities as well as different processing and measuring methods. It is clear that, different chemical composition can result in different nutritive value (Maheri-Sis *et al.*, 2007; 2008).

**Fig. 1:**
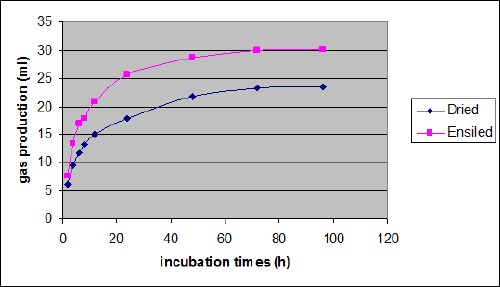
Gas production volume of dried and ensiled pomegranate seeds in different incubation times

Gas production volumes (ml/200mg DM) from *in vitro* incubation of pomegranate seed samples at different incubation times are illustrated in Table 2 and Figure 1.

**Table 2:** Gas production volume (ml/200mg DM) of dried and ensiled pomegranate seeds at different incubation times

Incubation times (h)	Dried	Ensiled	*P* value	S.E.M
2	6.20	7.67	0.0184	0.2702
4	9.45	13.46	0.0001	0.1108
6	11.79	16.90	0.0005	0.3462
8	13.18	17.84	0.0002	0.2458
12	15.12	20.90	0.0004	0.3660
24	17.91	25.76	0.0001	0.3660
48	21.87	28.65	0.0004	0.4400
72	23.34	29.97	0.0001	0.3099
96	23.49	30.12	0.0001	0.3116

Results indicate that ensiling leads to significant increase in gas production of pomegranate seeds at all incubation times. The gas volume at 24 h incubation, were 25.76 and 17.91 ml/200mg DM for ensiled and dried pomegranate seeds, respectively.

Sommart *et al*. (2000) demonstrated that gas volume is a good parameter for predicting digestibility, fermentation end products and microbial protein synthesis of the substrate by rumen microbes in the *in vitro* system.

Mirzaei-Aghsaghali *et al.*, (2011) reported that gas volume after 24 h incubation of dried pomegranate seed was 22.90 ml/200mg DM. Amount of gas produced at 24 h incubation is the most important criteria for estimating ME, NEL, OMD and SCFA; because of its high positive correlation by the nutritive value of feedstuffs (Menke and Steingass, 1988). However, different gas production in various studies can be due to different chemical constituents in the feed tested, animal types and breeds and quality of inoculum source (Menke and Steingass, 1988; Getachew *et al.*, 2004; Maheri-Sis *et al.*, 2008).

Gas production parameters (*a, b, c*) and estimated values of OMD, ME, NEL and SCFA of dried and ensiled pomegranate seeds are shown in Table 3.

**Table 3:** *In vitro* gas production parameters and estimated metabolisable energy (ME), net energy for lactation (NEL), Organic matter digestibility (OMD) and short chain fatty acids (SCFA) of dried and ensiled pomegranate seeds

Items	Dried	Ensiled	*P* value	S.E.M
*a* (ml)	5.23	4.84	0.6554	0.5668
*b* (ml)	17.98	24.62	0.0023	0.6820
*a+b* (ml)	23.20	29.46	0.0426	0.3283
*c* (ml/h)	0.0643	0.0930	0.0002	0.0007
ME (MJ/Kg DM)	5.10	6.37	0.0001	0.0496
NEL (MJ/Kg DM)	3.56	4.43	0.0001	0.0368
OMD (%)	34.62	43.15	0.0001	0.3246
SCFA (mmol)	0.3680	0.5553	0.0001	0.0008

*a*: the gas production from soluble fraction (ml/200mg DM)

*b*: the gas production from insoluble but fermentable fraction (ml/200mg DM)

*c*: rate constant of gas production during incubation (ml/h)

*a* + *b*: the potential gas production (ml/200mg DM)

Although there are no significant differences between gas production of soluble fraction (*a*) in ensiled and dried pomegranate seeds, insoluble but fermentable fraction (*b*), potential gas production (*a+b*) and rate constant of gas production (*c*) of treatments were significantly higher for ensiled than that of dried samples.

Our data is in agreement with Mirzaei-Aghsaghali *et al.*, (2011); based on their reports, amount of gas production parameters i.e. *a*, *b*, *a*+*b* and *c* were 5.69, 49.12, 54.18 ml/200 mg DM and 0.0141 ml/h, respectively.

Valizadeh *et al*. (2009) found that ensiling caused decrease in rumen degradation of pistachio by-products (which is tannin rich by-product similar to pomegranate by-products). Blummel and Becker (1997) suggest that the soluble fraction (*a*) of feeds makes it easily attachable by rumen microbes and resulted in more gas production.

Also the gas volume at asymptote (*b*) is an important index for predicting feed intake. Thus, it can be concluded that ruminants need to consume higher amount of ensiled than dried pomegranate seeds. Higher amount of gas production as well as rate of gas production in ensiled compared to dried samples may be affected by carbohydrate fractions (NFC), which are readily available to microbial fermentation. Maheri-Sis *et al*. (2008) suggested that different chemical components of feeds (e.g. Starch, NFC, OM, CP, NDF and soluble sugars contents) can result in different levels of in *vitro* gas production.

They have found that decreasing NDF and ADF and increasing NFC content of feedstuffs clearly results in higher *in vitro* gas production. Therefore, higher NFC and lower NDF and ADF content of ensiled pomegranate seeds may be major reason for the difference in gas production and consequent nutritive value.

The organic matter digestibility (OMD), metabolizable energy (ME), net energy for lactation (NEL) and short chain fatty acids (SCFA) of ensiled pomegranate seeds were significantly higher in comparison with that of dried samples (43.15%, 6.37 MJ/kg DM, 4.43 MJ/kg DM, 0.5553 mmol for ensiled samples vs. 34.62%, 5.10 MJ/kg DM, 3.56 MJ/kg DM, 0.3680 mmol for dried samples, respectively). Results of the current research agree with findings of Mirzaei-Aghsaghali *et al.*, (2011), who reported that estimated amounts of OMD, ME and SCFA of dried pomegranate seeds were 42.34%, 6.20 MJ/kg DM and 0.504 mmol, respectively.

Feizi *et al*. (2005) determined that digestibility of DM, OM, CP, CF, EE, energy, NFE, total digestible nutrients (TDN) and digestible OM content in DM of pomegranate seed were 44.8, 44.7, 62.5, 21.5, 38.3, 45, 61.5, 45.9 and 48.4%, respectively. Blummel *et al*. (1999) stated that gas volumes were produced quantitatively and qualitatively as a result of SCFA production (the amount of fermentative CO_2_ and CH_4_ could be accurately calculated from the amount and proportion of acetate, propionate and butyrate present in the incubation medium). Therefore increasing amount of SCFA had led to an increase in gas production which resulted in increased digestion and energy value.

In ruminants, acetate, propionate and butyrate (as predominant SCFAs), are readily absorbed and assimilated as a nutrient source. The SCFA account for between 50-70% of digestible energy intake (Mirzaei-Aghsaghali *et al.*, 2011). Between half and two-third of the total metabolizable energy may originate from these volatile fatty acids (VFA) produced from fermentative degradation by microorganisms in the rumen. Type of VFA produced is highly dependent on the type of diet, on the type of substrate fermented, and on the fermentation conditions (Bannink, 2007).

## Conclusion

The results of the current study based on chemical composition, OMD, ME, NEL and SCFA indicates that ensiling increases the nutritive value of pomegranate seeds and it is a better preservation method than drying for this by-product. Moreover ensiled pomegranate seeds have a higher nutritive value than dried for ruminants under *in vitro* conditions. However there is a need for *in vivo* studies to support the *in vitro* findings.
